# Assessment of hearing function and prevalence of hearing impairment among stroke patients and its relationship to leisure and social activity limitation: a nationwide population-based study

**DOI:** 10.25122/jml-2025-0019

**Published:** 2025-08

**Authors:** Amal Mohammed Sunyur, Mohamed Abdel-Haleem, Abdulaziz Khalid Alawfi, Ahmad Badawi, Layan Abdulaziz Alharbi, Hussam Abduljabbar Alahmadi, Sulaiman Abdullah, Zakaria Yahya Khawaji

**Affiliations:** 1 College of Medicine, Taibah University, Al-Madinah Al-Munawwarah, Saudi Arabia; 2 Department of Otolaryngology-Head and Neck Surgery, Faculty of Medicine, Taibah University, Al-Madinah Al-Munawwarah, Saudi Arabia

**Keywords:** stroke, quality of life, hearing impairment, post-stroke recovery

## Abstract

Stroke, which is a leading cause of disability, is presumed to affect hearing by impacting the auditory pathways or hearing organs, influencing communication and socialization. We analyzed data from the National Health Interview Survey (NHIS) from 2015–2018, including 118,625 individuals. Hearing function and activity limitations were evaluated by self-reported measures. Various statistical tests and regression analyses were used to compare and investigate the data between the different groups. Stroke patients had a higher prevalence of hearing dysfunction, with 23.8% reporting excellent hearing versus 46.9% among non-stroke individuals (*P* < 0.001). Additionally, stroke patients exhibited higher rates of little trouble hearing (19.9% vs. 10.9%), moderate trouble hearing (10.5% vs. 4.2%), much trouble hearing (8.7% vs. 2.2%), and deafness (0.8% vs. 0.3%) when compared to non-stroke subjects (*P* < 0.001 for all). Several factors were identified as significant contributors to hearing impairment among stroke patients, including male gender (OR = 1.58; 95% CI, 1.39–1.8; *P* < 0.001), diabetes (OR = 1.2; 95% CI, 1.04–1.38; *P* = 0.01), smoking (OR = 1.44; 95% CI, 1.2–1.73; *P* < 0.001), and sinusitis (OR = 1.45; 95% CI, 1.23–1.7; *P* <0.001). Stroke among subjects with hearing impairment was strongly related to limitation in leisure and social activities (OR = 2.5; 95% CI, 2.30–2.84). Our study demonstrates significantly higher rates of hearing impairment among stroke patients compared to non-stroke individuals, which adversely impacts their participation in leisure and social activities.

## INTRODUCTION

Stroke is an emergency medical condition and is the most common cause of disability worldwide and in the United States. It is estimated that approximately 800,000 people in the United States are affected by stroke annually [1]. Stroke can affect the auditory pathway at any level, potentially leading to hearing dysfunction. Post-stroke hearing loss can result from a stroke affecting the vertebrobasilar territory, damaging the brainstem, or from cortical damage, which is less frequent. Additionally, it is reported that hearing impairment after a stroke could result from damage to the hearing organ itself or the peripheral nerve, both of which are supplied by the internal auditory artery, a branch of the anterior inferior cerebellar artery. Population-based studies on hearing loss after stroke indicate that it is rather common, and a history of stroke increases the incidence of having hearing dysfunction. This, in turn, may impact the patient’s ability to communicate and limit their activity [2]. Furthermore, hearing loss can impact communication between the patient and the healthcare provider, potentially resulting in less efficient care provision and impacting rehabilitation [3].

Hearing loss after stroke has been reported to occur more often in elderly patients, men with tinnitus, lacunar strokes, bilateral multifocal strokes, and patients with arterial hypertension [4]. In a case-control study by Koohi *et al*., a combination of peripheral and central hearing loss was found to be the most common pattern of hearing loss in stroke patients [5]. A study conducted in India observed significant hearing impairment in patients after a stroke [3]. Moreover, a study in Taiwan concluded that stroke patients had higher rates of sensorineural hearing loss than non-stroke subjects [6].

Data on post-stroke auditory deficits is still lacking, as this topic has not been thoroughly researched to date. This may delay the development of proper screening methods and rehabilitative interventions for managing auditory dysfunction after stroke [2].

### Aims and objectives

1. To assess hearing function in stroke patients and compare it with that of non-stroke individuals.
2. To estimate the prevalence of hearing dysfunction among individuals with a history of stroke and identify contributing factors.
3. To evaluate the impact of hearing dysfunction on leisure and social activities in both stroke and non-stroke groups.

## MATERIAL AND METHODS

### Study design and population

**Figure 1:**
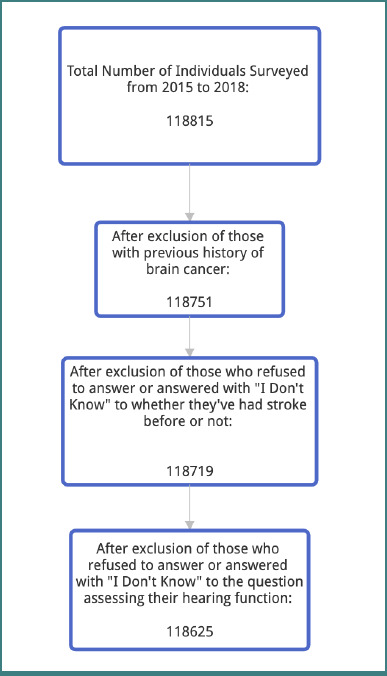
Flowchart of study inclusion and exclusion

This is a retrospective analysis of data from the National Health Interview Survey (NHIS). The NHIS is a large interview-based, nationally representative annual survey that collects health-related information from adults in the United States using a multistage clustered sampling technique to improve public health [7]. Data from the time range of 2015–2018 were used in this study, during which the questions asked of the participants were consistent. Data from the chosen time period was approved by the National Center for Health Statistics (NCHS). Informed consent in this study was established in accordance with Beth Israel Deaconess Medical Center policy. Hearing function and activity limitations were evaluated in two groups of patients: stroke patients and non-stroke patients. Those who refused to answer or answered either “not ascertained” or “don’t know” to the questions “Have you ever been told you have a stroke?” or “WITHOUT the use of hearing aids or other listening devices, is your hearing excellent, good, a little trouble hearing, moderate trouble, a lot of trouble, or are you deaf?” were excluded from the study (Figure 1). Furthermore, to minimize bias, patients with a previous history of structural brain disease, namely brain cancer, were also excluded. All other participants were included in the analysis (*n* = 118,625 subjects).

### Variables of the study

#### Dependent variable (outcome)

Hearing function, which is the dependent variable, was assessed by asking the participant, “WITHOUT the use of hearing aids or other listening devices, is your hearing excellent, good, a little trouble hearing, moderate trouble, a lot of trouble, or are you deaf?”. Those who responded with the following statements were considered to have hearing dysfunction: "a little trouble hearing”, “moderate trouble”, “a lot of trouble”, and “deaf”. As for activity limitation, the participants were asked twelve questions about the difficulty of performing different activities. We only considered three questions that are related to leisure and social activities. The three questions asked to the participants were: "By yourself, and without using any special equipment, how difficult is it for you to...do things to relax at home or for leisure (reading, watching TV, sewing, listening to music)?” "By yourself, and without using any special equipment, how difficult is it for you to...go out to things like shopping, movies, or sporting events?”; and "By yourself, and without using any special equipment, how difficult is it for you to...participate in social activities such as visiting friends, attending clubs and meetings, going to parties?”. Answers to each of these questions were either “Not at all difficult”, “only a little difficult”, “somewhat difficult”, “very difficult”, “can’t do at all”, or “Do not do this activity”.

#### Independent variable (exposure of interest)

Participants were classified as either stroke or non-stroke subjects. Those who answered “yes” to the question “Have you ever been told you had a stroke?” were considered to be stroke subjects, while those who answered “no” were considered to be non-stroke subjects. During the interview, demographic covariates (sex, marital status, and age) were also gathered by asking the following questions, respectively: “Are you male or female?”, “Are you now married, widowed, divorced, separated, never married, or living with a partner?”, and age as a continuous variable. As for clinical covariates, we considered the following comorbidities: diabetes, hypertension, hypercholesterolemia, and sinusitis based on the subjects’ answers to the questions stating whether or not they have ever been told they have diabetes, hypertension, high cholesterol, or sinusitis, and obesity based on the body mass index (BMI) of the subjects. Since it is recorded as a continuous variable, those who had a BMI of more than 25 were considered to be overweight, and those with a BMI of more than 30 were considered to be obese. As for smoking, which was assessed by directly asking the participants about their smoking status, those who answered “current every day smoker”, “current some day smoker”, or “smoker, current status unknown” were considered to be current smokers. Those who answered with either “former smoker”, “never smoker”, or “unknown if smoker” were categorized separately.

### Statistical analysis

An evaluation was conducted on the clinical and demographic data. Categorical data were compared using the chi-square test and are shown as percentages. The Shapiro-Wilk test was applied to assess the normality of continuous variables. The *t*-test was applied to compare normally distributed data, which are shown as means (with standard deviations). When presenting non-normally distributed data, medians along with interquartile ranges were used. The Mann-Whitney U test was applied for comparison. To investigate the significant association between the predictors and outcomes, multivariate logistic regression analyses were applied to assess the independent effects of multiple predictors on the binary outcome based on all variables that were significant in the descriptive analysis screening. Stata 15.1 was used for the statistical analysis (Stata Corp LLC, Texas, USA), Python (Python Software Foundation, version 3.8), and the Matplotlib library (Hunter, 2007) were used for data visualization.

## RESULTS

Table 1 displays the baseline characteristics of both stroke and non-stroke subjects. This study involved a total of 118,625 subjects, with 4,350 of them grouped as stroke cases and the other 114,265 grouped in the non-stroke group. The stroke group had a significantly higher mean age than the non-stroke group. Gender showed a similar distribution in both groups. Furthermore, a significant difference was observed in the rates of comorbid conditions, including hypertension, hypercholesterolemia, and diabetes mellitus, between the stroke group and the non-stroke group. However, rates of smoking were higher among the stroke group compared to the non-stroke group.

**Table 1:** Baseline characteristics of stroke and non-stroke populations

	Total	Stroke	Non-stroke	*P* value
**Number (%)**	118,625	4,360 (3.68)	114,265 (96.33)	
**Gender (male, *n* [%])**	53,589 (45.2)	1,992 (45.7)	51,597(45.2)	0.51
**Age (years, mean ± SD)**	51.21 ± 18.05	67.06 ± 13.97	50.14 ± 18.36	<0.001
Hypertension* (Yes, *n* [%])	41,920 (35.3)	3,262(74.8)	38,658(33.8)	<0.001
Hypercholesterolemia** (Yes, *n* [%])	36,071 (30.4)	2657 (60.9)	33,414 (29.2)	<0.001
Diabetes Mellitus status***				
Borderline (Yes, *n* [%])	2,972 (2.5)	179 (0.2)	2,794 (2.4)	<0.001
Non-diabetic (Yes, *n* [%])	102,727 (86.6)	2,873 (65.9)	99,854 (87.4)	
Diabetic (Yes, *n* [%])	12,874 (10.9)	1,306 (29.9)	11,568 (10.1)	
**Smoking status**				
Former smoker (Yes, *n* [%])	28,736 (24.2)	1,575 (36,1)	27,161 (23.8)	<0.001
Never smoker (Yes, *n* [%])	70,960 (59.8)	1,927 (44.2)	69,033 (60.4)	
Smoker (Yes, *n* [%])	18,533 (15.6)	844 (19.3)	17,689 (15.5)	
**Quality of hearing**				
Excellent (Yes, *n* [%])	54,634 (46.1)	1,037 (23.8)	53,597 (46.9)	<0.001
Good (Yes, *n* [%])	42,090 (35.5)	1,582 (36.3)	40,508 (35.5)	
Little trouble of hearing (Yes, *n* [%])	13,354 (11.3)	867 (19.9)	12,487 (10.9)	
Moderate trouble of hearing (Yes, *n* [%])	5,275 (4.4)	460 (10.5)	4,815 (4.2)	
A lot of trouble of hearing (Yes, *n* [%])	2,921 (2.5)	380 (8.7)	2,541 (2.2)	
Deaf (Yes, *n* [%])	331 (0.3)	34 (0.8)	297 (0.3)	

Hearing dysfunction was also noted to be more prevalent in the stroke group than the non-stroke group, with only 23.8% of the stroke group reporting excellent hearing as compared to 46.9% of the non-stroke group (*P* < 0.001). Interestingly, higher rates of good hearing were reported among the stroke group when compared to the non-stroke subjects. Little trouble hearing, moderate trouble hearing, a lot of trouble hearing, and being deaf were more prevalent in stroke patients compared to non-stroke subjects. In general, those who did not have hearing impairment (reported excellent or good hearing function) were more prevalent among the non-stroke group (82.4%) compared to subjects in the stroke group (60.1%). In contrast, those classified as having hearing impairment (reported little trouble hearing, moderate trouble hearing, a lot of trouble hearing, and being deaf) were higher in the stroke group than the non-stroke group (39.9% vs. 17.6%).

**Figure 2:**
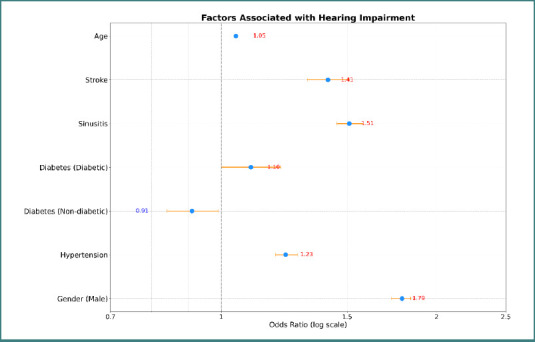
Forest plot of factors associated with hearing impairment

Table 2 shows the baseline characteristics of subjects with and without hearing impairments. 21,881 of the total subjects had hearing impairment, in contrast to 96,724 individuals who did not have hearing impairment. A higher mean age was reported in the hearing dysfunction group as compared to the non-hearing dysfunction group. Hearing impairment was also associated with higher rates of comorbid conditions, including hypertension, hypercholesterolemia, and diabetes mellitus. Furthermore, never smokers were higher in the non-hearing dysfunction group compared to the hearing dysfunction group. When compared in terms of stroke occurrence, those with hearing dysfunction had a higher rate of history of stroke compared to those without hearing dysfunction. The multivariate analysis shown in Figure 2 identified multiple factors that were significantly associated with hearing impairment. Increasing age was identified as a factor increasing the odds of developing hearing impairment. Furthermore, several comorbid conditions were identified to increase the odds of hearing impairment, including hypertension, having a history of sinusitis, having a history of stroke, and diabetes.

**Table 2:** Baseline characteristics of individuals with and without hearing dysfunction

	Total	Hearing dysfunction	Non-hearing dysfunction	*P* value
**Number (%)**	118,605	21,881 (18.45)	96,724 (81.55)	
**Gender (male, *n* [%])**	53,579 (45.2)	11,714 (53.5)	41,865 (43.3)	<0.001
**Age (years, mean ± SD)**	50.76± 18.5	63.43 ± 16.32	47.89 ± 17.74	<0.001
Hypertension* (Yes, *n* [%])	41,909 (35.3)	12,130 (55.4)	29,779 (30.8)	<0.001
Hypercholesterolemia** (Yes, *n* [%])	36,064 (30.4)	10,596 (48.4)	25,468 (26.3)	<0.001
Diabetes Mellitus status***				
Borderline (Yes, *n* [%])	2,972 (2.5)	804 (3.7)	2,168 (2.2)	<0.001
Non-diabetic (Yes, *n* [%])	102,709 (86.6)	16,902 (77.2)	85,807 (88.7)	
Diabetic (Yes, *n* [%])	12,872 (10.9)	4,164 (19)	8,708 (9)	
**Smoking status**				
Former smoker (Yes, *n* [%])	28,729 (24.2)	7,867 (36)	20,762 (21.6)	<0.001
Never smoker (Yes, *n* [%])	70,951 (59.8)	10,183 (46.5)	60,768 (62.8)	
Smoker (Yes, *n* [%])	18,531 (15.6)	3,761 (17.2)	14,770 (12.5)	
**Stroke (Yes, *n* [%])**	4,360 (3.7)	1,741(8)	2,619 (2.7)	<0.001

Table 3 compares the characteristics of those with and without hearing impairment among stroke and non-stroke patients. In the stroke group, hearing impairment was associated with a higher mean age compared to those without hearing impairment. Similarly, the mean age was also higher in the hearing dysfunction group among non-stroke patients. Higher rates of hypertension, hypercholesterolemia, and diabetes were also observed among stroke patients with hearing impairment compared to those without hearing impairment. Similar trends were also noted among non-stroke patients, with hearing dysfunction individuals having a higher prevalence of hypertension, hypercholesterolemia, and diabetes mellitus.

**Table 3:** Baseline characteristics of hearing dysfunction and non-hearing dysfunction groups stratified by stroke status

	Stroke			Non-stroke		
	**Hearing impairment**	**Non-hearing impairment**	***P* value**	**Hearing impairment**	**Non-hearing impairment**	***P* value**
**Gender (male, *n* [%])**	890 (51.1)	1,102 (42.1)	<0.001	10,824 (53.7)	40,763 (43.4)	<0.001
**Age (years, mean ± SD)**	71.18 ± 12.22	64.32 ± 14.39	<0.001	62.76 ± 16.46	47.43 ± 17.61	<0.001
Hypertension* (Yes, *n* [%])	1,350 (77.8)	1,910 (73)	<0.001	10780 (53.6)	27,869 (29.6)	<0.001
Hypercholesterolemia** (Yes, *n* [%])	1,135 (65.7)	593 (34.4)	<0.001	9,461 (47.2)	23,946 (25.5)	<0.001
Diabetes Mellitus status***						
Non-diabetic (Yes, *n* [%])	1,096 (63)	1,775 (67.9)	<0.001	15,806 (78.5)	84,032 (89.3)	<0.001
Borderline (Yes, *n* [%])	69 (4)	110 (4.2)		735 (3.7)	2,058 (2.2)	
Diabetic (Yes, *n* [%])	576 (33)	730 (27.9)		3,588 (17.8)	7,978 (8.5)	
**Smoking status**						
Former smoker (Yes, *n* [%])	693 (39.8)	882 (33.7)	<0.001	7,174 (35.6)	19,980 (21.2)	<0.001
Never smoker (Yes, *n* [%])	716 (41.1)	1,210 (46.2)		9,467 (47)	59,558 (63.3)	
Smoker (Yes, *n* [%])	326 (18.7)	518 (19.8)		3,435 (17.1)	14,252 (15.1)	
Chronic sinusitis (Yes, *n* [%])	382 (22)	479 (18.3)	0.003	1,351(17.4)	10,605 (11.3)	<0.001

A multivariate analysis of the factors associated with hearing dysfunction among stroke and non-stroke patients is shown in Table 4. Male sex was associated with higher odds of hearing dysfunction in both groups. A history of sinusitis and diabetes also emerged as significant predictors in both stroke and non-stroke patients. Smoking was a significant additional factor, increasing the likelihood of hearing dysfunction across both groups. Interestingly, hypertension and hypercholesterolemia were significant contributors only among non-stroke individuals, but not among those with stroke.

**Table 4:** Multivariate analysis of factors associated with hearing impairment in stroke and non-stroke groups

	Hearing impairment							
	Stroke				Non-stroke			
	OR	CI		*P* value	OR	CI		*P* value
**Gender (male)**	1.58	1.39	1.8	<0.001	1.71	1.65	1.77	<0.001
**Age**	1.04	1.038	1.05	<0.001	1.048	1.047	1.049	<0.001
**Hypertension (Yes)**	0.98	0.84	1.15	0.81	1.23	1.18	1.27	<0.001
**Hypercholesterolemia (Yes)**	1.13	0.98	1.3	0.09	1.26	1.2	1.3	<0.001
**Diabetes Mellitus status**								
Non-diabetic	Reference							
Borderline	1	0.73	1.4	0.97	1.1	1	1.2	0.051
Diabetic	1.2	1.04	1.38	0.01	1.21	1.15	1.27	<0.001
**Smoking status**								
Never smoker	Reference							
Former smoker	1.13	0.98	1.31	0.09	1.4	1.35	1.46	<0.001
Smoker	1.44	1.2	1.73	<0.001	1.66	1.58	1.73	<0.001
**Chronic sinusitis (Yes)**	1.45	1.23	1.7	<0.001	1.48	1.42	1.55	<0.001

**Figure 3:**
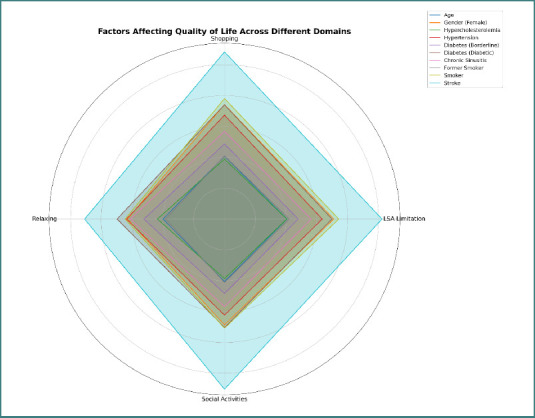
Radar chart of quality-of-life domains affected by hearing impairment

As shown in Table 5, female sex was consistently associated with limitations in leisure and social activities among individuals with hearing impairment. Stroke was strongly associated with such limitations overall and across all three domains: shopping, relaxation, and social activities (Figure 3).

**Table 5:** Factors associated with decreased quality of life among individuals with hearing impairment

	LSA limitation			Shopping			Relaxing			Social activities		
	OR	CI		OR	CI		OR	CI		OR	CI	
**Age**	1.01	1.01	1.02	1.02	1.02	1.02	.99	.99	1.00	1.01	1.01	1.02
**Gender (female)**	1.77	1.67	1.89	1.86	1.73	1.99	1.58	1.43	1.73	1.71	1.60	1.84
**Hypercholesterolemia (yes)**	1.01	0.95	1.08	0.97	0.91	1.05	1.09	0.99	1.20	0.97	0.89	1.04
**Hypertension (yes)**	1.57	1.48	1.70	1.68	1.56	1.81	1.59	1.43	1.77	1.56	1.44	1.69
**Diabetes mellitus**												
Non-diabetic	Reference											
Borderline	1.20	1.02	1.40	1.22	1.03	1.44	1.31	1.04	1.64	1.21	1.01	1.45
Diabetic	1.75	1.62	1.89	1.85	1.71	2.01	1.74	1.57	1.94	1.76	1.61	1.92
**Chronic sinusitis (yes)**	1.41	1.30	1.52	1.42	1.31	1.54	1.53	1.38	1.70	1.42	1.30	1.55
**Smoking status**												
Never smoker	Reference											
Former smoker	1.05	0.98	1.13	1.03	0.95	1.11	.96	.86	1.06	1.03	.95	1.11
Smoker	1.86	1.70	2.03	1.96	1.78	2.15	1.61	1.43	1.82	1.77	1.60	1.96
Stroke (yes)	2.56	2.30	2.84	2.71	2.42	3.03	2.27	1.99	2.59	2.76	2.46	3.09

## DISCUSSION

The study included both stroke and non-stroke populations, comprising a total of 118,625 subjects with varying baseline characteristics such as sex, age, comorbidities, smoking status, and hearing quality. Gender was predominantly female in both groups. Moreover, rates of non-smokers were higher in the non-stroke group compared to the stroke group. This can be explained by the strong association between smoking and stroke incidents. As shown by another study, there is a non-linear dose response between the risk of stroke and smoking; a very small dose of cigarette smoking can have a similar risk to a large dose [8].

Also, our study shows that hearing dysfunction is more prevalent among stroke patients compared to the non-stroke group, who reported having excellent or good hearing function. Among stroke patients, 19.9% reported a little trouble hearing, 10.5% reported moderate trouble, 8.7% reported a lot of trouble, and 0.8% were deaf. These findings further support the higher rate of hearing dysfunction among stroke patients. Similar results were reported in another study, which found that stroke patients had twice the incidence of sudden sensorineural hearing loss (SSNHL) compared with non-stroke patients, corresponding to a 71% increased risk of hearing dysfunction in the stroke group [6].

Among the study subjects, 21,881 had hearing impairment and 96,724 did not. The hearing impairment group showed a higher association with other comorbid conditions like age, hypertension, hypercholesterolemia, diabetes mellitus, smoking, and stroke history. Similar findings have been reported in previous studies, which confirmed the association between hearing loss and comorbidities, emphasizing that the severity of hearing loss is primarily influenced by the severity of these underlying conditions [9-11]. Older age was strongly associated with hearing impairment, as the affected group had a higher mean age compared with those without hearing dysfunction. This finding is consistent with previous research showing that the incidence of hearing impairment increases with advancing age and has wide-ranging adverse effects on patients’ social, mental, and physical well-being. In severe cases, this may lead to cochlear implantation as the treatment of choice [12].

In this study, hearing impairment was influenced by a variety of general factors, including male gender, age, and the presence of comorbidities like a history of stroke, sinusitis, hypertension, or diabetes, highlighting that these factors play a significant role in hearing dysfunction. A study conducted in China yielded similar findings regarding the influence of gender, age, and increased blood pressure on hearing dysfunction [13]. This consistency across different populations amplifies the idea that these factors are universally significant. Furthermore, higher rates of hearing loss in males compared to females—irrespective of stroke history— are reported multiple times in the literature, suggesting that male gender is a significant predictor of hearing impairment [6,14].

Stroke emerged as a significant risk factor for hearing dysfunction in our study. The risk appeared even more pronounced when stroke coexisted with other factors such as male sex, older age, and hypertension. This implies that stroke may not only be a standalone risk factor but could worsen the impact of hearing impairment when accompanied by other pre-existing comorbidities. A cross-sectional study echoed these findings, confirming that stroke is indeed a significant risk factor for hearing impairment, especially when it is combined with factors such as male gender, old age, and hypertension [4]. In contrast, a study found no significant difference in hearing impairment between stroke and non-stroke patients, which contrasts with our results and those of other investigations [5]. As far as we are concerned, this discrepancy could be due to differences in population characteristics or study design. Moreover, an increase in hearing impairment in smokers compared to nonsmokers was demonstrated in multiple cross-sectional studies [15-17]. The association between smoking and hearing impairment, though less emphasized in some studies, should not be ignored. Smoking might act as an intensifying factor, aggravating the effects of other risk factors.

Also, we found multiple factors associated with increasing hearing dysfunction in both stroke and non-stroke patients, which are male gender, sinusitis, diabetes, and smoking. Moreover, hypertension and hypercholesterolemia both contributed to the increasing odds of having hearing dysfunction only in non-stroke patients. Przewoźny *et al*. reported similar findings, identifying male sex and hypertension as significant factors associated with hearing impairment in ischemic stroke patients. On the other hand, factors like diabetes mellitus (DM), smoking, and hyperlipidemia had no significant association with hearing impairment [4]. Another study found that both men and women were significantly associated with hearing loss in stroke patients compared to non-stroke patients; however, men had a higher HR than women (adjusted HR 1.78 and 1.67, respectively) [6].

Hearing dysfunction in general is associated with older age, male gender, comorbidities like DM, smoking, and cardiovascular risk factors, which are consistent with our findings [18]. In addition, sinusitis and other otorhinolaryngological conditions may contribute to various forms of hearing impairment, including conductive, sensorineural, or mixed types. These factors influence hearing impairment in the general population and are not specific to stroke, which is consistent with our observations [19]. The literature, however, is lacking in terms of studying specific factors like smoking, otorhinolaryngological conditions, and hyperlipidemia in stroke patients and their relation to hearing impairment.

Stroke showed the strongest association with decreased quality of life (QoL) among patients with hearing impairment, contributing to limitations in leisure and social activities (LSA), including shopping, relaxation, and social interaction. Other factors associated with decreased QoL are age, DM, smoking, hypertension, female sex, and sinusitis. These findings are consistent with a study assessing QoL and social interactions in elderly male patients with different levels of hearing impairment that found that all patients with hearing impairment had a greater risk of social isolation (OR = 3.29; 95% CI, 2.38-4.54) among those using hearing aids but still unable to hear. In addition, men who could not hear had significantly higher odds of reporting a decrease in QoL domains (pain/discomfort, mobility problem, anxiety/depression), with the highest prevalence observed in men who could not hear despite a hearing aid (pain/discomfort [OR = 2.68 (1.92–3.75)]; mobility problems [OR = 2.65 (1.93–3.64)]; anxiety/depression [OR = 1.79 (1.24–2.59)] [20].

In general, QoL in stroke patients is decreased and further deteriorates with advancing age. When additional comorbidities such as hearing impairment are present—as shown in our study—a further decline in QoL can be expected [21]. An important strength of our study is the large sample size, which exceeds that of previous investigations on this topic. Furthermore, and contrary to other studies, we accounted for multiple confounding factors in our analysis.

### Limitations

The results of this study should be interpreted in accordance with its potential limitations. First, this study was done by retrospectively analyzing cross-sectionally gathered data, which, despite being the best method for wide-scale estimation of hearing function among stroke patients, only permits the identification of associations but cannot establish temporality or causality. Furthermore, self-report was used to gather the data for this study, which means that there is a possibility of recall bias. Also, measuring hearing function and activity limitation was done based on the subjective assessment of the participants rather than objective methods of estimating hearing function or the degree of activity limitation, which may affect the accuracy of the provided information. Thus, the interpretation of the results related to the two categories “hearing impairment” and “non-hearing impairment” should be done relatively. Another limitation is that some uncollected confounding factors may be related to hearing impairment or social and leisure activity limitations that were not considered in this study. The fact that the results of this study on the effect of stroke on hearing function and their relationship to activity limitation align with previously published studies on this matter strengthens the confidence in the conclusions in this study.

## CONCLUSION

In conclusion, our study showed a significantly higher prevalence of hearing impairment among stroke patients compared to non-stroke subjects. The relationship between stroke and hearing impairment is shown, with stroke patients having increased odds of deteriorating hearing function, affecting their ability to participate in leisure and social activities. Therefore, the findings of this study highlight the importance of regular and routine hearing impairment screening in stroke patients, which may contribute to more effective rehabilitative measures and improve the quality of life of these patients.

## Data Availability

NHIS data are publicly available on the agency website for no charge.
